# Heavy Strength Training in Older Adults: Implications for Health, Disease and Physical Performance

**DOI:** 10.1002/jcsm.13804

**Published:** 2025-04-16

**Authors:** Tiril Tøien, Ole Kristian Berg, Roberto Modena, Mathias Forsberg Brobakken, Eivind Wang

**Affiliations:** ^1^ Department of Health and Social Sciences Molde University College Molde Norway; ^2^ Sport Mountain and Health Research Center (CeRiSM) University of Verona Verona Italy; ^3^ Department of Psychosis and Rehabilitation Psychiatry Clinic St. Olavs University Hospital Trondheim Norway

**Keywords:** heavy resistance training, high load, intended velocity, maximal strength training, rehabilitation

## Abstract

Older adults typically exhibit reductions in skeletal muscle maximal strength and the ability to produce force rapidly. These reductions are often augmented by concomitant acute and chronic diseases, resulting in attenuated physical performance and higher propensity of falls and injuries. With the proportion of older adults in the population increasing, there is an alarming need for cost‐effective strategies to improve physical performance and combat a multitude of age‐related diseases. Surprisingly, despite convincing evidence emerging over three decades that strength training can substantially improve maximal strength (1RM), rate of force development (RFD) and power, contributing to improved health, physical performance and fall prevention, it appears that it has not fully arrived at the older adults' doorsteps. The aim of the current narrative review is to accentuate the convincing benefits of strength training in healthy and diseased older adults. As intensity appears to play a key role for improvements in 1RM, RFD and power, this review will emphasize training performed with heavy (80%–84% of 1RM) and very heavy loads (≥ 85% of 1RM), where the latter is often referred to as maximal strength training (MST). MST uses loads of ~90% of 1RM, which can only be performed a maximum of 3–5 times, 3–5 sets and maximal intentional concentric velocity. Strength training performed with loads in the heavy to very heavy domain of the spectrum may, because of the large increases in muscle strength, focuses on neural adaptations and relatively low risk, provides additional benefits for older adults and contrasts current guidelines which recommend low‐to‐moderate intensity (60%–70% of 1RM) and slow‐moderate concentric velocity. This review also provides information on practical application of MST aimed at practitioners who are involved with preventive and/or rehabilitative health care for older adults.

## 1 The Ageing Human and Muscle Strength

Over the past decades, the relative proportion and life expectancy of older adults (> 60 years) has risen considerably, resulting in increased disability rates and healthcare needs [1]. Ageing is characterized by higher likelihood of numerous diseases, such as cardiovascular disease (CVD), pulmonary diseases, cancer, musculoskeletal diseases, neuromuscular diseases and sarcopenia (historically, typically referred to as the loss of skeletal muscle mass) [2, 3], leading to increased physical inactivity and immobilization [4]. Unfortunately, this potentially has major impact on skeletal muscle strength, which is closely related to the reduced physical function commonly observed with ageing [4]. As the noted demographic changes represents a great societal and economic challenge, there is an urgent need for tactics to alleviate the burden placed upon the healthcare system. Strength training has large potential to be one such strategy, as it may aid the ageing population to stay healthy and independent for longer.

Specifically, low muscle strength can predict future mobility limitations [5] and risk of falls and fractures [6] and is associated with reduced physical performance [7]. Importantly, high muscle strength is strongly and independently associated with reduced risk of all‐cause mortality [8, 9, 10, 11] and mortality from cancer [12]. Maximal muscle strength, often expressed as the heaviest external weight that can be lifted successfully once (1RM), or maximal isometric force (MVC), declines gradually from young adulthood to old age, with a distinct acceleration of loss around the sixth decade of life [1]. However, the decrease is less pronounced in upper versus lower extremities [2]. Interestingly, the ability to produce force rapidly, commonly referred to as rate of force development (RFD) and expressed as [Δforce/Δtime], and skeletal muscle power (the product of force and contraction velocity [force × velocity]) [13, 14] declines on a steeper trajectory than the loss of muscle strength [3, 4, 13]. The age‐related muscle strength deterioration not caused by neurological or muscular disease is also typically referred to as dynapenia [15]. Collectively, low 1RM, RFD and power pose substantial challenges for health and physical performance with old age [7, 14, 15].

## 2 Strength Training and Standard Guidelines for Healthy and Diseased Older Adults

Strength training is systematic training aimed to improve maximal and/or rapid force production (i.e., 1RM and RFD). It is characterized from low to high relative intensity (low: < 70%; moderate: 70%–79%; heavy: 80%–84%; and very heavy: ≥ 85% of 1RM), with the latter implying loads lifted with few repetitions and rest periods lasting 3–4 min or more [5, 6, 7, 8]. Thus, the terms heavy‐very heavy strength training and high‐intensity strength training are sometimes used interchangeably. Notably, a meta‐analysis of strength training in older adults found no measurable strength improvements to be achieved below a relative training load of 50% of 1RM [9]. However, some strength gains may occur at low intensity if exercises are continued to the point of failure [10, 11, 12], although even 100 body weight squats per day for 4 months did not increase maximal strength in older adults, as intensity was likely too low [16]. This suggests that volume and intensity are not interchangeable variables. Above the threshold intensity, a dose–response relationship has been observed, suggesting heavy‐very heavy strength training (≥ 80% 1RM) to yield greater improvements in muscle strength [6, 17, 18, 19, 20, 21, 22]. A set conducted at ~90% of 1RM produces greater muscle activation than a set at 70% of 1RM [23], which likely is of importance to chronic adaptations in neuromuscular function. Indeed, superior effects have been observed for both 1RM and RFD following training at 85%–90% of 1RM of one leg compared to 60%–70% of 1RM for the other leg, within the same individuals [24]. Repetitions should preferably also be executed with maximal intended velocity (i.e., maximal mobilization of force) in the concentric phase to maximally stimulate the neural system during the contraction [25].

Despite the likely greater improvements in muscle strength following heavy‐very heavy strength training (80–84 and ≥ 85% of 1RM, respectively), guidelines for healthy older adults appear to recommend utilization of moderate‐intensity strength training [26, 27, 28, 29]. Similar guidelines also exist for patients with osteopenia or osteoporosis [30], heart conditions and hypertension [31, 32, 33], chronic obstructive pulmonary disease (COPD) [34] and cancer [35], where the recommended strength training intensity is ~60%–70% 1RM for ~10–15 repetitions and slow, controlled execution of movement. Recognizing numerous studies presenting evidence suggesting that healthy and diseased older adults may, and should, train with heavier loads than current guidelines, granting large enhancements in neuromuscular performance, should strength training guidelines be updated? And is it time strength training with heavy‐very heavy loads is implemented in clinical practice? The aim of the present narrative review is to explore literature examining the effects and feasibility of strength training using heavy‐very heavy loads in healthy and diseased older adults and provide hands‐on, practical guidelines for practitioners and individuals with detailed execution of exercises that should be recommended for this population.

## 3 Strength Training in the Ageing Healthy and Diseased Population

The first studies utilizing heavy strength training in an elderly population (> 65 years) were presented in the late 1980s [36] and early 1990s [37, 38]. In the hallmark studies by [37, 39], frail nursing home residents of almost 90 years old (mean age 87 years, range 72–98 years) performed strength training with heavy loads of 80% of 1RM, with considerable improvements in strength and functional status. Yet, to date, few older adults are even aware of the recommendations of incorporating muscle strengthening exercises in their weekly activity, and fewer yet perform strength training with heavy loads [40, 41, 42]. Reasons for this may certainly be multifactorial. Explanations may include the older population's unfamiliarity with strength training, that they assume recreational aerobic activities (e.g., walking, cycling, Pilates, swimming or yoga) to be sufficient for increased strength or that it is unsafe to utilize heavy loads [42].

## 4 Low‐Moderate Versus Heavy‐Very Heavy Strength Training

**FIGURE 1:**
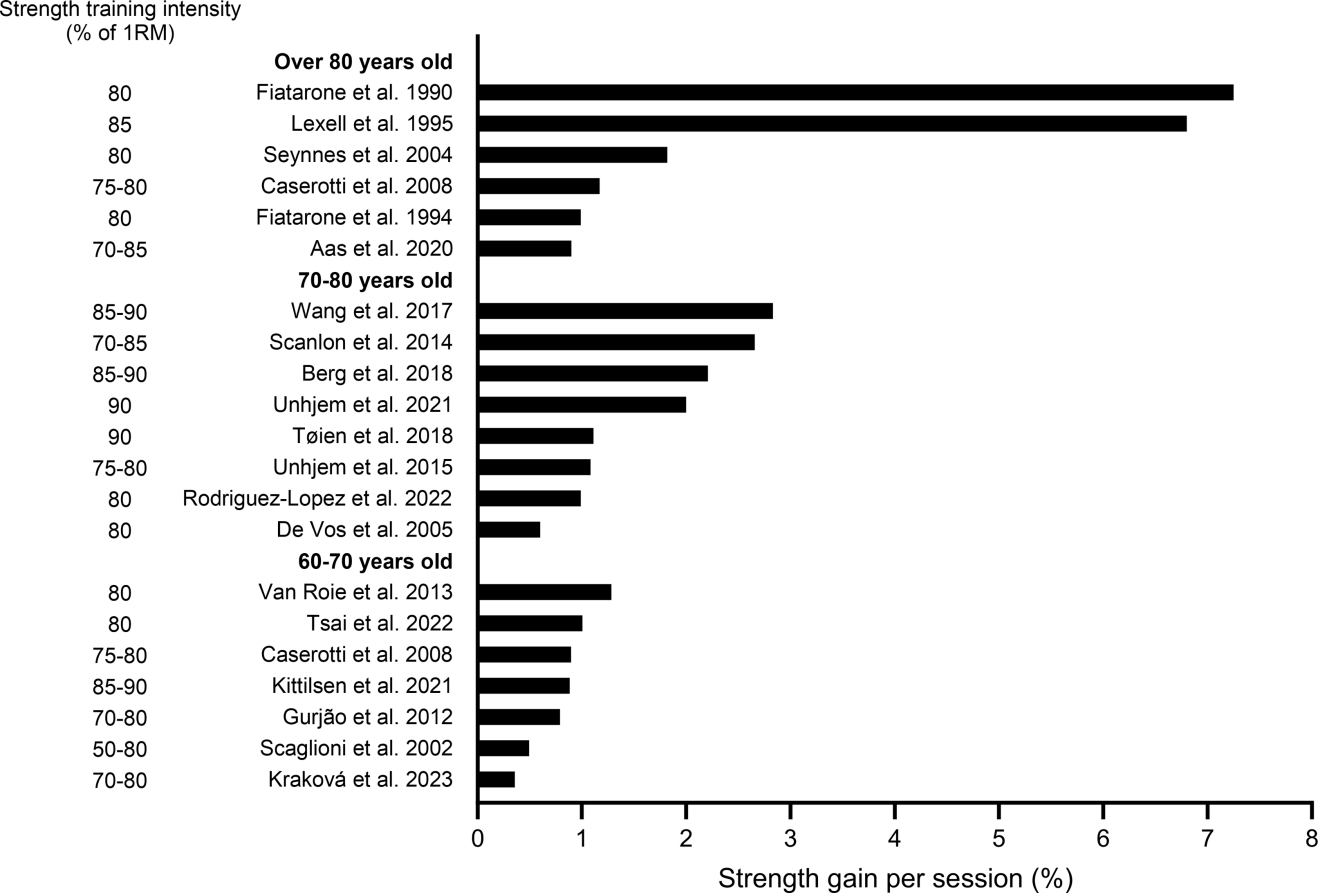
Lower extremity maximal strength gain per session (%) in older adults (> 60 years) following heavy‐very heavy strength training (80–84 and ≥ 85% of one repetition maximum, respectively).

Older adults are consistently documented to exhibit substantial improvements following heavy‐very heavy strength training, with increases of ~0.5%–8.5% in maximal force (1RM or isometric maximal voluntary contraction; MVC) per strength training session (Figure 1). This results in an impressive average increase of ~2.5% per session [6, 10, 17, 19, 21, 22, 37, 38, 39, 43, 44, 45, 46, 47, 48, 49, 50, 51, 52, 53, 54]. Importantly, maximal force improvements in the included studies indicate that the largest effect sizes are observed following heavy‐very heavy strength training compared to low‐moderate strength training [6, 39, 45, 46]. This is supported by a previous study with an intra‐individual design, in young adults, where very heavy training matched for volume (total weight lifted) increased strength ⁓40% more compared to moderate training [24]. Notably, even with the very heavy loads, few repetitions and limited time expenditure, it is also shown to yield similar improvements in size and proportion of Type II muscle fibres as moderate strength training, despite a lower volume [6]. However, although there are several indications that heavy‐very heavy strength training yields more strength gain in older adults [55, 56, 57], a direct comparison between training loads is challenging because control of training volume is warranted. In addition, improvements are dependent on baseline strength, such that the largest gains physiologically (e.g., kg) and mathematically (%) are expected in the most untrained individuals with lowest strength. In fact, this was observed in frail and very weak nursing home residents [37, 38].

The improvements in maximal force are indeed remarkable, especially when considering that the yearly age‐related decline in maximal strength from the fifth decade is about 1% per year. Implying, that, for example, a 70‐year individual may restore maximal muscle strength to the level of young individuals after only a few weeks of training. Adding weight to the evidence of great strength training–induced responses with advancing age, one study showed similar increases in dynamic leg press 1RM in older, middle aged, and younger adults following 8 weeks of very heavy strength training [21]. Together, these findings suggest that heavy‐very heavy strength training for older individuals/patient groups should, at the very least, be included in the relevant guidelines.

**FIGURE 2:**
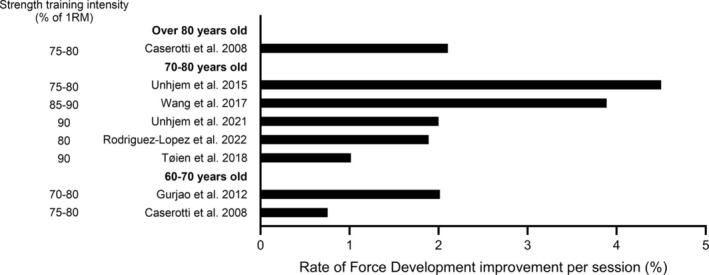
Lower extremity rate of force development gain per session (%) in older adults (> 60 years) following heavy‐very heavy strength training (80–84 and ≥ 85% of one repetition maximum, respectively).

The ability to develop force rapidly is considered a key feature of muscle strength and is of critical functional importance at higher age [58]. In older adults, RFD is reported to increase by 1%–4.5% per strength training session following heavy‐very heavy strength training (Figure 2) [6, 22, 44, 47, 50, 53, 54]. Accordingly, increases in maximal muscle power of 0.5%–1% per session is reported [37, 59]. It should be noted that despite the functional relevance of RFD and maximal muscle power, relatively few studies have examined these variables in healthy older adults following strength training.

## 5 Strength Training in Frail and Diseased Populations: Is It Safe?

Strength training with heavy‐very heavy loads, near 1RM, in frail and diseased individuals may seem counterintuitive and unsafe. However, this assumption is unsupported by the literature. In the last three decades, with a convincing number of publications in recent years, studies have demonstrated that heavy strength training in frail and diseased populations is safe and highly effective to improve strength outcomes and functional performance (see Table S1: https://doi.org/10.6084/m9.figshare.25211180) [60, 61, 62, 63, 64, 65], even among the oldest‐old [66]. It is important to note that loads are relative to the individual's maximal strength, and as such, in frail and diseased populations, 90% of 1RM may only amount to leg press or squat training with loads similar, or even less than, an individual's bodyweight in the beginning of a training period [64, 67]. In such cases, performing strength training with sufficient load is of upmost importance to decrease the overall load during everyday tasks.

Importantly, if the heavy‐very heavy loads and maximal intended velocity of movement is limited to the concentric phase during the execution of repetitions, the risk of injuries is considerably reduced [68]. Much like when performing a squat jump, the risk of damage is not high when force is being developed, but rather on the time of impact during the landing [69]. In contrast, if the eccentric phase is fast, such as for a counter movement jump, forces may also be quite substantial in the turning phase [70]. Therefore, heavy‐very heavy strength training should be performed with a slow, controlled eccentric movement phase and preferably a controlled pause in the movement prior to concentric action but can safely include maximal intended velocity in the concentric phase. This is the explanation why very heavy strength training, few repetitions, and maximal intended velocity in the concentric phase has proven safe and feasible, even in very frail patient populations such as women with osteoporosis or osteopenia [71], directly following hip fracture surgery [67], cancer patients undergoing adjuvant therapy [72] and stroke survivors [73]. In fact, in these studies, the very heavy load was even performed from the first training session and onwards.

In patients with CVD, strength training per se may appear counterintuitive due to concerns about potential adverse events following high haemodynamic pressure; however, Fan et al. [74] observed that combined endurance and strength training is more beneficial for aerobic and skeletal muscle performance than endurance training alone in stable or/and treated CVD patients. Importantly, as blood pressure builds with each repetition, patients with medically stable coronary artery disease experience increased haemodynamic response from rest to both few repetitions with very heavy loads (4RM) and more repetitions with lower loads (15RM) [75], albeit to substantially greater extent after the 15RM training. The application of upper versus lower extremity training modality may also be considered, with the latter typically producing somewhat less haemodynamic response than the former [76]. However, taken together, the data suggest that heavy‐very heavy strength training results in a lower cardiovascular risk than strength training with more repetitions and should therefore be the training modality of choice for this patient population.

## 6 Neuromuscular Adaptations Following Strength Training

Interestingly, with advancing age, maximal muscle strength and RFD decline at a considerably greater rate than muscle mass [4, 77, 78, 79] because the nervous system play a pivotal role [13, 80]. Force production is modulated through efferent neural drive to the skeletal muscle, that is, motor unit recruitment and the rate at which the motor units can be recruited [81]. Thus, a main target to prevent strength loss due to age and/or disease should be to maintain or increase efferent neural drive. Large increases in efferent neural drive have been observed following heavy and very heavy strength training [22, 44]. An adaptation that is not present following unloaded ballistic training, for example, plantar flexion [47]. Even in patients with neurological diseases, such as multiple sclerosis (MS) and Parkinson's disease, increased efferent neural drive has been documented following very heavy strength training [82, 83]. Importantly, contrary to many older adults' beliefs [42], recreational activity is not sufficient to preserve efferent neural drive, likely due to the limited involvement of fast motor units. Strength training appears to be imperative [84, 85].

Heavy‐very heavy strength training has been consistently reported to counteract the loss of muscle cross sectional area and muscle volume in older adults [36, 37, 86, 87]. Both Type I [36, 38, 86] and Type II fibre areas are documented to increase, even after relatively short training interventions of only a few months [6, 36, 38, 86]. Interestingly, a preferential increase in Type II fibre area was observed by Wang et al. [6], following very heavy strength training in older adults. Moreover, as older adults typically experience specific atrophy of Type II fibres, this response normalized older adults compared to young adults in terms of area‐specific muscle fibre distribution. Type II fibres have a higher intrinsic force and RFD [88]. Thus, the observed gains in Type II fibre area would seem highly beneficial to optimize training‐induced gains in and/or maintenance of maximal strength and RFD with increasing age. A complete reversal of the muscle architecture changes occurring with age appear to be possible following strength training, such as increased pennation angle, fibre fascicle length and tendon stiffness [89, 90, 91]. Altogether, these improvements in the nervous, muscular and tendinous systems likely explain the enhanced physical performance observed following heavy‐very heavy strength training.

## 7 Clinical Implications for Strength Training

### 7.1 Functional Importance of Strength Training

The functional relevance of a high 1RM, RFD and power is particularly strong for older adults in force‐demanding tasks such as chair rising and stair climbing [92, 93]. Rising from a chair is crucial for independence in everyday life [94] and when impaired may pose a serious limitation with detrimental consequences to physical and mental health. At some point declining muscle strength becomes a serious impediment for activities of daily living, as it eventually may result in individuals not being able to get up from a seated position without aid [95]. Heavy‐very heavy strength training can help maintain 1RM and RFD with increasing age, or even restore it to a level where it is no longer as debilitating a factor in everyday life, for both healthy and diseased older adults [73, 82, 92, 96]. Maintenance of strength and thus functional status throughout life is also important for healthy individuals, as it allows continuation of their youthful pattern to be maintained, thus preserving a high‐quality life for longer.

## 8 Strength Training, Fall Avoidance and Postural Stability

Maintaining or improving muscle strength is important for postural stability and prevention of falls [97]. This is relevant for trained and untrained older adults. For the untrained, it limits everyday life through avoidance of activity due to fear of falling [98] and could cause life‐threatening fractures at old age [99]. For the trained, it may be important for being able to perform exercise as well as recreational and sporting activities. Fall situations and many physical activities are characterized by time‐restricted conditions, where there is less time to produce the necessary force for posture correction (< 200 ms) [100], compared to what is normally needed to achieve maximal contraction force (> 300 ms) [101]. In line with this notion, fallers appear to have lower RFD than non‐fallers [102]. Notably, heavy‐very heavy strength training has proven to enhance the RFD in this crucial early phase of force production [44, 65]. Likely, maximal intended velocity in the concentric phase of strength training movement is important to maximize this adaptation due to the great involvement of the nervous system. Indeed, very heavy strength training with maximal intended velocity in the concentric phase has been observed to improve postural stability in frail hip fracture patients [67].

## 9 Strength Training and Walking Work Efficiency

Several studies have documented a reduced oxygen cost of locomotion, following very heavy strength training, across a wide array of patient populations [5, 72, 103, 104] as well as for healthy older adults [6]. This impact on aerobic endurance performance is linked to improved mechanical efficiency of submaximal muscle contractions in the trained musculature [7, 105]. Thus, activities such as walking at a given speed will become relatively easier. Alternatively, the speed can be increased without increasing the overall experienced exertion in relation to before very heavy strength training.

## 10 Application of Strength Training in Clinical Practice

Heavy‐very heavy strength training is documented to be an excellent and safe strength training format to maximally mitigate the loss of force in healthy older adults and numerous patient populations. These principles of strength training can easily be applied to different target muscles based on an individual's or a patient specific limitations and needs. Very heavy strength training may be performed using four sets at 4RM (i.e., a load that can only be lifted four times), with progressive loading to maintain the same relative intensity throughout the training period as strength increases. In practical terms, this means that if the individual is able to lift five repetitions or more, the resistance should be increased to only allow 4 repetitions in the next session. As the intensity is dependent on the individual's strength, the same principles can be used to improve the function of the frailest patients as well as maintain or improve performance in healthy older adults.

**FIGURE 3:**
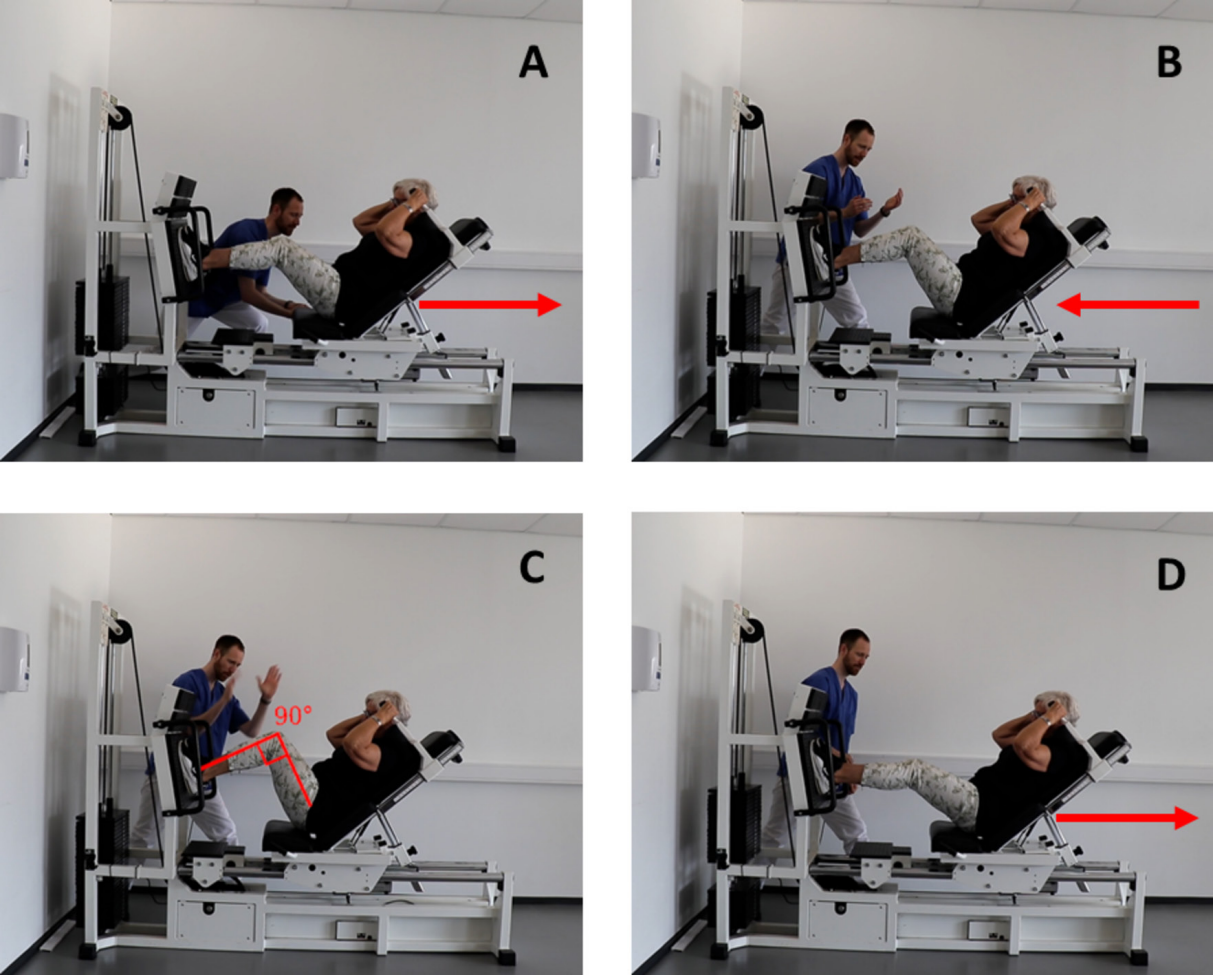
A description of the execution of very heavy strength training (maximal strength training [MST]) in a horizontal leg press apparatus. (A) Set the weight/resistance to ~90% of 1RM, which can only be lifted four times (i.e., 4RM). Assist the participant from the bottom position to knees near extended. Note the start position should be slightly below 90° angle in the knee joint so that the weights do not rest on the weight stack in the bottom position where the knee angle should be 90° (see Panel C). (B) Instruct the subject to conduct a slow controlled eccentric movement, lasting ~2–3 s. (C) Give a clear stop command when angle between tibia and femur is 90°, where a short stop in the movement should be emphasized before the next part of the movement. (D) Give a clear and encouraging command to lift the weight by extending the legs. The movement should be done with maximal intended velocity. Given the high load the actual movement velocity will be slow. Repeat steps B–D three more times for a total of four repetitions. Give the participant 3–4 min rest before the next set. This should be repeated for a total of four sets.

The exercise should start with a slow, controlled eccentric phase lasting ~2–3 s, followed by a short pause (~1 s), before initiating the concentric phase (see Figure 3). In the concentric phase, one should aim to move the weight as fast as possible (maximal intended velocity) [25]. To achieve this, the subject should be encouraged to try to perform the concentric contraction as explosively as possible, although, due to the heavy weight, the actual movement velocity will be slow. This is important to note, as prevalence of injuries may be related to uncontrolled fast movements, particularly in the eccentric phase [106]. Due to the very high load and maximal intended velocity, this way of performing very heavy strength training is also referred to as maximal strength training (MST).

**FIGURE 4:**
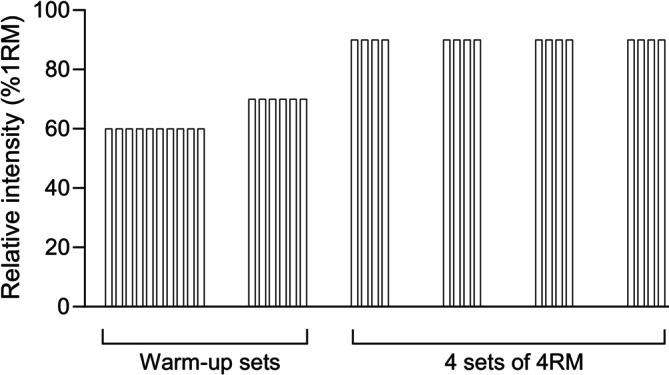
A typical training session of very heavy strength training (maximal strength training, MST). The sessions start with two warm‐up sets at a low‐moderate intensity, where 8–10 repetitions are performed in the first set and 6–8 repetitions in the final warm‐up set, before commencing with four training sets of four repetitions maximum (4RM). The training sets should be separated by 3–4 min of rest.

For exact progress and prescription of training load, one may want to assess 1RM prior to the exercise training. However, this is not necessary when using a set RM (e.g., 4RM) to limit the resistance. Moreover, 4RM can also be used to indicate an increase in maximal strength throughout a training period, as this is a weight corresponding to ~90% of 1RM [8]. This also allows the individual/patient to start training right away. A typical training session begins with a specific warm‐up in the exercise used by letting the individual warm‐up with ~8–10 repetitions at a light weight. Based on the relative ease of this first warm‐up weight, a moderate load is chosen, and the individual lifts another warm‐up set of ~6–8 repetitions, before commencing with four sets of 4RM. The training sets should be separated by about 3–4 min of rest. An example of a typical training session is presented in Figure 4. A video presentation of an MST set can be found in the Supporting Information (https://doi.org/10.6084/m9.figshare.23552085). In addition, strength training frequency may vary considerably between studies, and the optimum number be a question of some debate. However, 2–3 training sessions per week is typically applied in most of the included studies, with higher frequency associated with larger strength gains [107]. This results in a ≥ 48‐h break between training sessions, which appears sufficient for recovery [108] and safe for the participants, based on the reported studies.

## 11 Strength Training to Target the Individual or Patients' Specific Limitations

One general recommendation is to perform strength training of the lower extremities, as strength reduction is more affected in the lower compared to upper extremities with age [109]. Moreover, the lower extremities are crucial for locomotion during everyday activities. For this purpose, a horizontal leg press may be ideal. The leg press is preferred over a free weight squat, as technique will minimally limit the intensity of the load. However, the exercise prescription should consider the main challenges of the specific disease or individual. For instance, for patients with osteoporosis or osteopenia, which is defined by a low bone mineral density and impaired bone quality of the spine and hip [110], axial loading through the spine should be included to stimulate bone density enhancement. Hack squat or horizontal leg press, where the back can be reclined to ensure loading through the spine, is recommended to attain axial loading while at the same time limit the impact of technique on using very heavy loads [71, 111].

Although, leg press can generally be recommended, the principles of heavy‐very heavy strength training can be employed to any exercise or targeted muscle group. Importantly, some diseases or injuries may require other exercises to target an affected or impaired muscle or muscle group. Examples may include bench press for wheelchair users [112], dorsiflexion for droop foot patients or leg abduction following hip surgery [67, 113]. Lastly, an intriguing observation is that unilateral very heavy strength training may induce adaptations in the untrained, contralateral limb [22]. This principle may be utilized in acute or chronic periods of immobilization to limit the loss of function in the immobilized limb.

## 12 Conclusion

Despite numerous studies documenting the effectiveness of heavy‐very heavy strength training to mitigate the age‐ and disease‐related decline in muscle strength, the application in clinical practice seems to be underutilized. Notably, heavy‐very heavy strength training has the potential to reverse several decades of age‐related decline in 1RM, RFD and power in both healthy and diseased older adults. The current review highlights the positive effects of heavy‐very heavy strength training for healthy and diseased older adults and the feasibility, safety, and clinical implications of such exercise. Specifically, strength training with heavy‐very heavy loads should be recommended for all older adults and implemented in patient care, especially of the lower extremities as the strength loss is more pronounced in the locomotor muscles with age. This review also gives practical instructions on how to conduct a session of strength training, using very heavy loads, few repetitions and maximal intended velocity in the concentric phase.

## Conflicts of Interest

The authors declare no conflicts of interest.
