# Correction to ‘Heavy Strength Training in Older Adults: Implications for Health, Disease and Physical Performance’

**DOI:** 10.1002/jcsm.70014

**Published:** 2025-07-27

Tøien, T. , Berg, O. , Modena, R. , Brobakken, M. and Wang, E. (2025), Heavy Strength Training in Older Adults: Implications for Health, Disease and Physical Performance. Journal of Cachexia, Sarcopenia and Muscle, 16: e13804, 10.1002/jcsm.13804.40241440 PMC12003923

In paragraph 2 of the manuscript, some of the papers referred to in text were incorrect and the correct references not listed. The following paragraph includes the correct references for those statements and reference list.

Specifically, low muscle strength can predict future mobility limitations [1] and risk of falls and fractures [2] and is associated with reduced physical performance [3]. Importantly, high muscle strength is strongly and independently associated with reduced risk of all‐cause mortality [4, 5, 6, 7] and mortality from cancer [8]. Maximal muscle strength, often expressed as the heaviest external weight that can be lifted successfully once (1RM), or maximal isometric force (MVC), declines gradually from young adulthood to old age, with a distinct acceleration of loss around the sixth decade of life [9]. Albeit, the decrease is less pronounced in upper versus lower extremities [10]. Interestingly, the ability to produce force rapidly, commonly referred to as rate of force development (RFD), and expressed as [Δforce/Δtime], and skeletal muscle power (the product of force and contraction velocity [force × velocity]) [11, 12] declines on a steeper trajectory than the loss of muscle strength [12, 13, 14]. The age‐related muscle strength deterioration not caused by neurological or muscular disease is also typically referred to as dynapenia [15]. Collectively, low 1RM, RFD and power pose substantial challenges for health and physical performance with old age [3, 16, 17].

We apologize for this error.
